# Prognostic Nutritional Index (PNI) as a potential predictor and intervention target for perioperative ischemic stroke: a retrospective cohort study

**DOI:** 10.1186/s12871-023-02216-8

**Published:** 2023-08-10

**Authors:** Min Liu, Miao Sun, Ting Zhang, Peng Li, Jin Liu, Yanhong Liu, Yuxiang Song, Siyuan Liu, Huikai Yang, Zhikang Zhou, Dandan Chang, Guyan Wang, Weidong Mi, Yulong Ma

**Affiliations:** 1 Department of Anesthesiology, The First Medical Center of Chinese PLA General Hospital, Beijing, 100853 China; 2 Department of Anesthesiology, Beijing Tongren Hospital, Capital Medical University, Beijing, 100730 China; 3 Nation Clinical Research Center for Geriatric Diseases, Chinese PLA General Hospital, Beijing, 100730 China; 4 Department of Anesthesiology, The First Affiliated Hospital, Jinzhou Medical University, Jinzhou, 121000 China; 5 Department of Anesthesiology, The Sixth Medical Center of PLA General Hospital, Beijing , 100048 China; 6 Hangzhou Le9 Healthcare Technology Co., Ltd, Hangzhou, 311200 China; 7 Department of Anesthesiology, Affiliated Hospital of Nantong University, Nantong, 226001 China

**Keywords:** Prognostic Nutritional Index (PNI), Perioperative ischemic stroke, Postoperative complication, Predictor, Intervention target

## Abstract

**Background:**

The prognostic nutritional index (PNI) is a nutritional indicator and predictor of various diseases. However it is unclear whether PNI can be a predictor of perioperative ischemic stroke. This study aims to evaluate the association of the preoperative PNI and ischemic stroke in patients undergoing non-cardiac surgery.

**Methods:**

The retrospective cohort study included patients who underwent noncardiac surgery between January 2008 and August 2019. The patients were divided into PNI ≥ 38.8 and PNI < 38.8 groups according to the cut-off value of PNI. Univariate and multivariate logistic regression analyses were performed to explore the association between PNI and perioperative ischemic stroke. Subsequently, propensity score matching (PSM) analysis was performed to eliminate the confounding factors of covariates and further validate the results. Subgroup analyses were completed to assess the predictive utility of PNI for perioperative ischemic stroke in different groups.

**Results:**

Amongst 221,542 hospitalized patients enrolled, 485 (0.22%) experienced an ischemic stroke within 30 days of the surgery, 22.1% of patients were malnourished according to PNI < 38.8, and the occurrence of perioperative ischemic stroke was 0.34% (169/49055) in the PNI < 38.8 group. PNI < 38.8 was significantly associated with an increased incidence of perioperative ischemic stroke whether in univariate logistic regression analysis (OR = 1.884, 95% CI: 1.559—2.267, *P* < 0.001) or multivariate logistic regression analysis (OR = 1.306, 95% CI: 1.061—1.602, *P* = 0.011). After PSM analysis, the ORs of PNI < 38.8 group were 1.250 (95% CI: 1.000–1.556, *P* = 0.050) and 1.357 (95% CI: 1.077–1.704, *P* = 0.009) in univariate logistic regression analysis and multivariate logistic regression analysis respectively. The subgroup analysis indicated that reduced PNI was significantly associated to an increased risk of perioperative ischemic stroke in patients over 65 years old, ASA II, not taking aspirin before surgery, without a history of stroke, who had neurosurgery, non-emergency surgery, and were admitted to ICU after surgery.

**Conclusions:**

Our study indicates that low preoperative PNI is significantly associated with a higher incidence of ischemic stroke in patients undergoing non-cardiac surgery. Preoperative PNI, as a preoperative nutritional status evaluation index, is an independent risk factor useful to predict perioperative ischemic stroke risk, which could be used as an intervenable preoperative clinical biochemical index to reduce the incidence of perioperative ischemic stroke.

**Supplementary Information:**

The online version contains supplementary material available at 10.1186/s12871-023-02216-8.

## Introduction

Perioperative ischemic stroke is an under-recognized but life-threatening complication of surgery resulting in high rates of disability and mortality [1]. Epidemiologic data suggests that postoperative stroke occurs approximately between 0.1% and 9.7% of patients undergoing surgery [2]. The risk of death is 26% for patients after general surgery, which could increase to up to 60% in those who previously had a stroke, and perioperative ischemic stroke is associated with a high mortality rate of patients [3, 4]. Due to delays in obtaining diagnostic imaging, narrow thrombolysis time window, and the reduced use of thrombolysis given the risk of recent surgery, perioperative ischemic stroke is often associated with poor outcomes. Therefore, it is crucial to identify the optimal risk factors to predict perioperative ischemic stroke risk, so as to intervene in time and actively provide treatment to reduce the incidence of perioperative ischemic stroke in clinical practice.

Emerging evidence shows that inflammation is key in the pathophysiology of the development, acute damage cascades, and chronic course after ischemic stroke in clinical and animal studies [5]. The chronic inflammatory process, caused by the persistence of factors that contribute to the inflammatory process, is a long-term physiological response to elements such as malnutrition, stress, viral infections, and environmental toxins [5]. Nutrition may play a central role in the regulation of chronic inflammation in perioperative ischemic stroke. Thus researching appropriate nutritional parameters is critical for evaluating the inflammatory process effectively in patients diagnosed with perioperative ischemic stroke.

The prognostic nutritional index (PNI), which is an index calculated by serum albumin concentration and lymphocyte value of peripheral blood, reflects both nutritional and inflammatory status, and prognosis in patients [6]. Unlike other screening tools, PNI can be easily evaluated in laboratory tests during preoperative diagnosis. To date PNI is generally used to evaluate the various cancers [7–9] and carprognosis of diovascular diseases [10–12]. High preoperative PNI might be a predictor of postoperative AKI and surgical prognosis in patients who underwent open hepatectomy for hepatocellular carcinoma [13]. In addition, PNI has been reported as a predictor of long-term mortality in older adults who had an ischemic stroke [14]. It’s worth noting that a large number of studies evaluating PNI have focused on disease outcomes such as survival and mortality, rather than predictors of disease occurrence. Besides, leucocyte-to-lymphocyte ratio (LLR) is an inflammatory factor which is closely associated with postoperative outcomes in patients with various clinical conditions [15]. However so far PNI has not been used to predict the risk of ischemic stroke in patients after surgery.

In this study, we hypothesized that the primary outcome is decreased preoperative PNI could be associated with the increase of perioperative ischemic stroke after non-cardiac surgery, and the secondary outcome is that lower preoperative PNI was significantly associated with a higher incidence of perioperative ischemic stroke in certain subgroups such as patients over 65 years old, neurosurgery, and postoperative ICU admission. This study aims to test the hypothesis and determine the threshold value of preoperative PNI for predicting the occurrence of perioperative ischemic stroke.

## Materials and methods

### Study design and patients

We retrospectively reviewed perioperative data for 221,542 hospitalized patients from the First Medical Center of Chinese PLA General Hospital from January 2008 to August 2019. The study included patients who underwent non-cardiac surgery with anesthesia. The exclusion criteria were the followings: (1) age < 18 years old, (2) duration of surgery ≤ 60 min, (3) regional anesthesia, (4) American Society of Anesthesiologists (ASA) classification ≥ IV, and (5) missing data for any confounders.

### Ethical approval

The study was following the Declaration of Helsinki and was approved by Ethics Committee of Chinese PLA General Hospital (No. S2021-496–01), and the date of the research proposal was 1 March 2022. The requirement of written informed consent for patients was waived by Ethics Committee of Chinese PLA General Hospital, because the study was retrospective and all data were anonymous.

### Definitions of outcomes

The primary outcome was defined as a diagnosis, in the 30 days following the surgery, of perioperative ischemic stroke, an episode of neurological dysfunction caused by focal cerebral, spinal, or retinal infarction, with imaging or other objective evidence of ischemia in a vascular distribution ultimately manifesting as motor, sensory, or cognitive impairment [16, 17]. We identified hospitalized patients who were billed for any ICD-9-CM/ICD-10-CM diagnosis of ischemic stroke within 30 postoperative days.

### Data collection

We used clinical data on perioperative variables from the electronic medical record system. Patient demographic data includes age, sex, body mass index (BMI), American Society of Anesthesiologists physical score (ASA), the presence of comorbid diseases —such as hypertension, diabetes mellitus, coronary heart disease, arterial fibrillation, arrhythmology, previous ischemic stroke, transient ischemic attack (TIA), chronic obstructive pulmonary disease (COPD), peripheral vascular disease, and renal dysfunction—, and preoperative mean arterial pressure (MAP). Preoperative medication use includes β-blockers, ACEI, ARB, aspirin, and glucocorticoid. Laboratory tests (in the 3 days prior to surgery) include hemoglobin, albumin, total bilirubin, and prothrombin time, as well as leucocyte-to-lymphocyte ratio (LLR) = total leucocyte count / total lymphocyte count, PNI = 10 × serum albumin (g/dL) + 0.005 × total lymphocyte count (per mm^3^) [18].

The intraoperative data are emergency status, type of surgery, malignant tumor surgery, duration of procedures, estimated blood loss, bleeding volume, crystalloid or colloid infusion, non-steroid anti-inflammatory drugs (NSAIDs) use, and use of glucocorticoid medication. In addition, data on ICU admission after surgery was also collected.

### Association between PNI and perioperative ischemic stroke

We used the receiver operating characteristic (ROC) curve to calculate the optimal cut-off value of PNI and LLR. The ROC analysis showed the optimal cut-off value of PNI should be set at 38.8 for predicting risk of perioperative ischemic stroke. Subsequently, we performed univariable and multivariable logistic analyses to evaluate the association between perioperative ischemic stroke and PNI as categorical variables. We adjusted multivariable logistic models for age, sex, hypertension, diabetes mellitus, prior ischemic stroke, β-blockers medication, aspirin medication, preoperative glucocorticoid, preoperative MAP, preoperative LLR, facility, surgical types, bleeding volume, and ICU admission after surgery.

### Propensity score‑matching analysis and adjustment

To address potential residual confounding, we performed propensity score-matching (PSM) to further assess the prognostic value of the PNI [19]. Propensity scores were calculated via a multivariate logistic regression analysis for baseline differences. Patients with PNI ≥ 38.8 and PNI < 38.8 were matched at a 2:1 ratio based on their propensity scores using nearest-neighbor matching, with a matching tolerance of 0.01%. We then used Kernel density plots of propensity scores to examine equivalence between matched patients. The baseline differences in clinical covariates between the PNI ≥ 38.8 and PNI < 38.8 groups were balanced using a PSM analysis. We calculated the standardized mean differences (SMD) to compare baseline differences before and after PSM, with minor differences being defined as an absolute value lower than 0.10 (small effect size).

### Subgroup analyses

We conducted subgroup analyses to explore the association between PNI and perioperative ischemic stroke in different subgroups organized by age, sex, ASA, aspirin, neurosurgery department, emergency status, ICU admission after surgery, and previous ischemic stroke. We generated a forest plot to summarize PNI predictions for perioperative ischemic stroke in subgroups.

### Statistical analysis

Continuous variables presented as mean (SD) or median (interquartile range, IQR) were compared using Student t test or Manne-Whitney U test as appropriate. Categorical variables presented as *n* (%) were compared using χ^2^ test or Fisher exact test.

To examine the association between preoperative PNI and perioperative ischemic stroke, we treated PNI as a categorical variable and conducted univariable and multivariable logistic regression analysis. The variables in the multivariable logistic regression analysis were those with statistically significant differences between groups in the univariate logistic regression analysis and in clinical practice. Results are presented as the odds ratio (OR) with the 95% confidence interval (CI). The optimal cut-off value of PNI was calculated according to the receiver operating characteristic (ROC) curve. To further reduce or eliminate the bias between the two groups (PNI ≥ 38.8 and PNI < 38.8), we performed a PSM analysis to further assess the prognostic value of the PNI using cut-offs in perioperative ischemic stroke. In PSM analysis, patients in the two groups were matched by propensity score (PS) at a 2:1 ratio. To further explore the association between PNI and perioperative ischemic stroke in different subgroups, we conducted subgroup analyses with multivariable logistic regression stratified by several key variables (age, sex, ASA, preoperative aspirin, neurosurgery department, emergency treatment, postoperative ICU, previous ischemic stroke).

The logistic regression model was performed using R version 4.0.1 (R Foundation, Vienna, Austria). Statistical significance was set at a two-tailed *P*-value < 0.05. Odds ratios with 95% confidence intervals (CI) were displayed for all models.

## Results

### Clinical characteristics of the study cohort

**Fig. 1:**
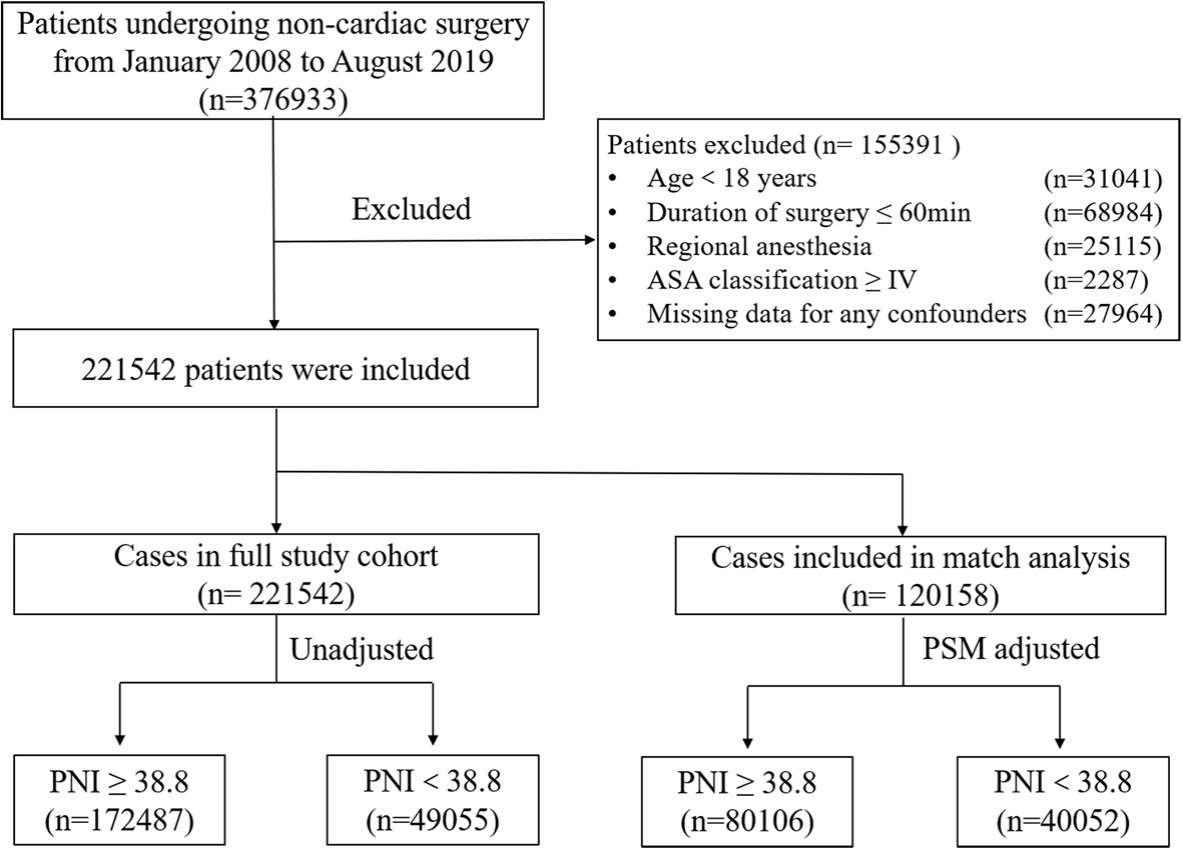
Study flow diagram. ASA, American Society of Anesthesiologists; PNI, prognostic nutritional index; PSM, propensity score matching

A total of 376,933 hospitalized patients undergoing non-cardiac surgery from January 2008 to August 2019 were enrolled in the study. After excluding 155,391 patients based on the described exclusion criteria, 221,542 eligible patients were enrolled in the analyses (Fig. 1). The median age of the enrolled patients was 52 years old (IQR: 41, 62), and 50.7% of the enrolled patients (112,315/221,542) were male. The incidence of perioperative ischemic stroke was 0.21% (485/221,542) in the overall cohort study.

### Baseline characteristics of patients

In order to accurately predict perioperative ischemic stroke risk, we completed the ROC curve, which showed the optimal cut-off value of PNI to be 38.8, with an area under the ROC curve of 0.589 (Supplementary Fig. 1), and the optimal cut-off value of LLR as 3.3, with an area under the ROC curve of 0.644 (Supplementary Fig. 2). The baseline characteristics of the patients are presented according to PNI ≥ 38.8 and PNI < 38.8 groups in Table 1. All baseline characteristics differed between PNI ≥ 38.8 and PNI < 38.8 groups except for preoperative ARB. Compared to patients in the PNI ≥ 38.8 group, patients in PNI < 38.8 group were more likely to be older, male, have a lower BMI, and showed more comorbidities (hypertension, diabetes mellitus, coronary heart disease, arterial fibrillation, arrhythmology, previous ischemic stroke, TIA, COPD, peripheral vascular disease, renal dysfunction) and longer ICU admissions after surgery. We also observed a trend of patients in PNI < 38.8 group having higher levels of preoperative LLR, surgery duration, blood loss, crystalloids and colloids infusion than patients in PNI ≥ 38.8 group.

**Table 1:** Baseline characteristics of unadjusted sample and propensity score-matched sample

Characteristic	Unadjusted sample (*n* = 221,542)			PSM adjusted (2:1) (*n* = 120,158)		
	**PNI ≥ 38.8 (*n* = 172,487)**	**PNI < 38.8 (*n *= 49,055)**	**SMD**	**PNI ≥ 38.8 (*n* = 80,106)**	**PNI < 38.8 (*n* = 40,052)**	**SMD**
Age, years	49 (14.2)	57 (13.9)	0.511	55 (13.3)	55 (13.6)	0.009
Sex (male) (%)	86,940 (50.4)	25,375 (51.7)	0.026	40,809 (50.9)	19,687 (49.2)	0.036
BMI, kg·m^2^	24.55 (3.66)	23.74 (3.68)	0.221	24.07 (3.41)	24.07 (3.71)	0.001
ASA classification (%)			0.366			0.137
Class I	28,342 (16.4)	4145 (8.4)		9523 (11.9)	4026 (10.1)	
Class II	133,422 (77.4)	37,340 (76.1)		63,467 (79.2)	30,848 (77.0)	
Class III	10,723 (6.2)	7570 (15.4)		7116 (8.9)	5178 (12.9)	
Hypertension (%)	33,189 (19.2)	10,453 (21.3)	0.051	18,347 (22.9)	7859 (19.6)	0.08
Diabetes mellitus (%)	20,111 (11.7)	7253 (14.8)	0.092	11,097 (13.9)	5454 (13.6)	0.007
Coronary heart disease (%)	5613 (3.3)	2395 (4.9)	0.082	3510 (4.4)	1685 (4.2)	0.009
Arterial fibrillation (%)	497 (0.3)	297 (0.6)	0.048	332 (0.4)	179 (0.4)	0.005
Arrhythmology (%)	25,622 (14.9)	8402 (17.1)	0.062	11,517 (14.4)	6424 (16.0)	0.046
Previous ischemic stroke (%)	3536 (2.1)	1521 (3.1)	0.066	2072 (2.6)	1018 (2.5)	0.003
Transient ischemic attack (%)	3933 (2.3)	1853 (3.8)	0.087	2289 (2.9)	1293 (3.2)	0.022
Chronic obstructive pulmonary disease (%)	1208 (0.7)	528 (1.1)	0.04	680 (0.8)	344 (0.9)	0.001
Peripheral vascular disease (%)	5586 (3.2)	2605 (5.3)	0.103	2867 (3.6)	2053 (5.1)	0.076
Renal dysfunction (%)	1227 (0.7)	813 (1.7)	0.088	716 (0.9)	626 (1.6)	0.061
Preoperative MAP, mmHg	91.9 (11.5)	91.1 (11.7)	0.066	92.8 (11.4)	91.1 (11.7)	0.153
Preoperative β blockers (%)	5903 (3.4)	2265 (4.6)	0.061	3215 (4.0)	1690 (4.2)	0.01
Preoperative ACEI (%)	3388 (2.0)	1043 (2.1)	0.011	1750 (2.2)	803 (2.0)	0.013
Preoperative ARB (%)	6824 (4.0)	1905 (3.9)	0.004	3582 (4.5)	1491 (3.7)	0.038
Preoperative aspirin (%)	4639 (2.7)	1683 (3.4)	0.043	2791 (3.5)	1276 (3.2)	0.017
Preoperative glucocorticoid (%)	10,737 (6.2)	4944 (10.1)	0.141	6187 (7.7)	3204 (8.0)	0.01
Preoperative Hemoglobin, g/L	137 (17.1)	122 (18.6)	0.823	135 (17.1)	123(18.2)	0.669
Preoperative LLR	3.48 (2.26)	4.67 (4.37)	0.343	3.93 (2.95)	4.08 (2.74)	0.051
Preoperative LLR						
< 3.28	106,753 (61.9)	20,760 (42.3)	0.399	39,339 (49.1)	19,621 (49.0)	0.002
≥ 3.28	65,734 (38.1)	28,295 (57.7)		40,767 (50.9)	20,431 (51.0)	
Emergency (%)	3527 (2.0)	1943 (4.0)	0.112	2120 (2.6)	1309 (3.3)	0.037
Surgical procedures (%)			0.43			0.023
Stomatological and ENT	27,047 (15.7)	4202 (8.6)		8005 (10.0)	4023 (10.0)	
Trauma surgery	4420 (2.6)	2096 (4.3)		2750 (3.4)	1474 (3.7)	
Gynaecology	12,413 (7.2)	2935 (6.0)		5411 (6.8)	2777 (6.9)	
Intra-abdominal surgery	51,148 (29.7)	22,750 (46.4)		32,445 (40.5)	15,894 (39.7)	
Joint arthroplasty	11,749 (6.8)	4126 (8.4)		7098 (8.9)	3605 (9.0)	
Spine	14,961 (8.7)	3232 (6.6)		6034 (7.5)	3034 (7.6)	
Urinary surgery	15,461 (9.0)	3076 (6.3)		5806 (7.2)	2908 (7.3)	
Neurosurgery	17,170 (10.0)	2707 (5.5)		5314 (6.6)	2622 (6.5)	
Thoracic or vascular	14,287 (8.3)	3077 (6.3)		5683 (7.1)	2950 (7.4)	
Other (plastic surgery, etc.)	3831 (2.2)	854 (1.7)		1560 (1.9)	765 (1.9)	
Malignant tumor surgery (%)	74,358 (43.1)	26,216 (53.4)	0.208	40,895 (51.1)	19,956 (49.8)	0.025
Duration of procedures, min	167.5 (96.1)	188.4 (102.1)	0.211	180.5 (101.2)	180.4 (98.2)	0.001
Estimated blood loss, mL	203.2 (403.4)	302.8 (565.5)	0.203	256.3 (485.7)	279.0 (523.2)	0.045
Bleeding volume						
≤ 50	71,316 (41.3)	13,925 (28.4)	0.303	25,274 (31.6)	12,565 (31.4)	0.008
(50,200]	64,971 (37.7)	19,842 (40.4)		32,417 (40.5)	16,133 (40.3)	
> 200	36,200 (21.0)	15,288 (31.2)		22,415 (28.0)	11,354 (28.3)	
Crystalloids infusion, ml/kg/min	1511.5 (740.6)	1756.4 (820.2)	0.313	1656.0 (795.2)	1661.9 (775.0)	0.007
Colloids infusion, ml/kg/min	492.7 (487.6)	652.7 (550.0)	0.308	578.3 (519.5)	622.7 (543.3)	0.084
Intraoperative NSAIDs (%)	46,315 (26.9)	14,785 (30.1)	0.073	23,170 (28.9)	11,574 (28.9)	0.001
Intraoperative glucocorticoid (%)	139,015 (80.6)	40,034 (81.6)	0.026	65,235 (81.4)	32,598 (81.4)	0.001
ICU admission after surgery (%)	17,906 (10.4)	8296 (16.9)	0.191	9886 (12.3)	4657 (11.6)	0.022

*Abbreviations: SMD* Standardized mean difference, *PSM* Propensity Score Matching, *PNI* Prognostic nutritional index, *BMI* Body mass index, *COPD* Chronic obstructive pulmonary disease, *ASA* American Society of Anaesthesiologists classification system, *ACEI* Angiotensin-converting enzyme inhibitors, *ARB* Angiotensin receptor blockers, *LLR* Leucocyte-to-lymphocyte ratio, *E.N.T* Otolaryngology head, and neck surgery, *MAP* Mean arterial pressure, *NSAIDs* Non-steroidal anti-inflammatory drugs

### Association between PNI and perioperative ischemic stroke

We initially explored the association between PNI as categorical variable and perioperative ischemic stroke using univariate and multivariate logistic analyses. The univariate analysis showed that age, hypertension, diabetes mellitus, prior ischemic stroke, β-blockers medication, aspirin medication, preoperative glucocorticoid, preoperative MAP, preoperative LLR, facility, surgical procedures, bleeding volume, and ICU admission after surgery were associated with a greater risk of perioperative ischemic stroke (Supplementary Table 1). The odds ratio (OR) of the PNI < 38.8 group was 1.884 (95% CI: 1.559—2.267, *P* < 0.001) in the univariate analysis, that is, the decreased PNI (PNI < 38.8) was significantly associated with an increased incidence of perioperative ischemic stroke (Table 2).

**Table 2:** Association between PNI with Perioperative Ischemic Stroke after non-cardiac surgery

Analysis method	OR	95%CI	*P*-value
Logistic regression analysis			
Model 1 (Univariate)	1.884	1.559—2.267	< 0.001
Model 2 (Multivariate)	1.306	1.061—1.602	0.011
Propensity score analysis			
Model PSM 1 (Univariate)	1.250	1.000–1.556	0.050
Model PSM 2 (Multivariate)	1.357	1.077–1.704	0.009

*PNI* Prognostic nutritional index, *OR* Odds ratio, *CI* Confidence interval, *PSM* Propensity score matching

Model 2 adjusted for age, sex, hypertension, diabetes mellitus, prior ischemic stroke, β-blockers medication, aspirin medication, preoperative glucocorticoid, preoperative MAP, preoperative LLR, facility, surgical procedures, bleeding volume, and ICU admission after surgery

Model PSM 1 was a univariate regression model

Model PSM 2 was a multivariate regression model

Statistically significant factors in univariate analysis were then included in the multivariate regression analysis to investigate the significant predictors of perioperative ischemic stroke. Patients in PNI < 38.8 group showed a higher OR of perioperative ischemic stroke (OR = 1.306, 95% CI: 1.061—1.602, *P* = 0.011). In addition, the variables that acted as independent risk factors for predicting perioperative ischemic stroke in the multivariate logistic regression analysis are shown in the Supplementary Table 1.

### Propensity score-matching analysis and adjustment

**Fig. 2:**
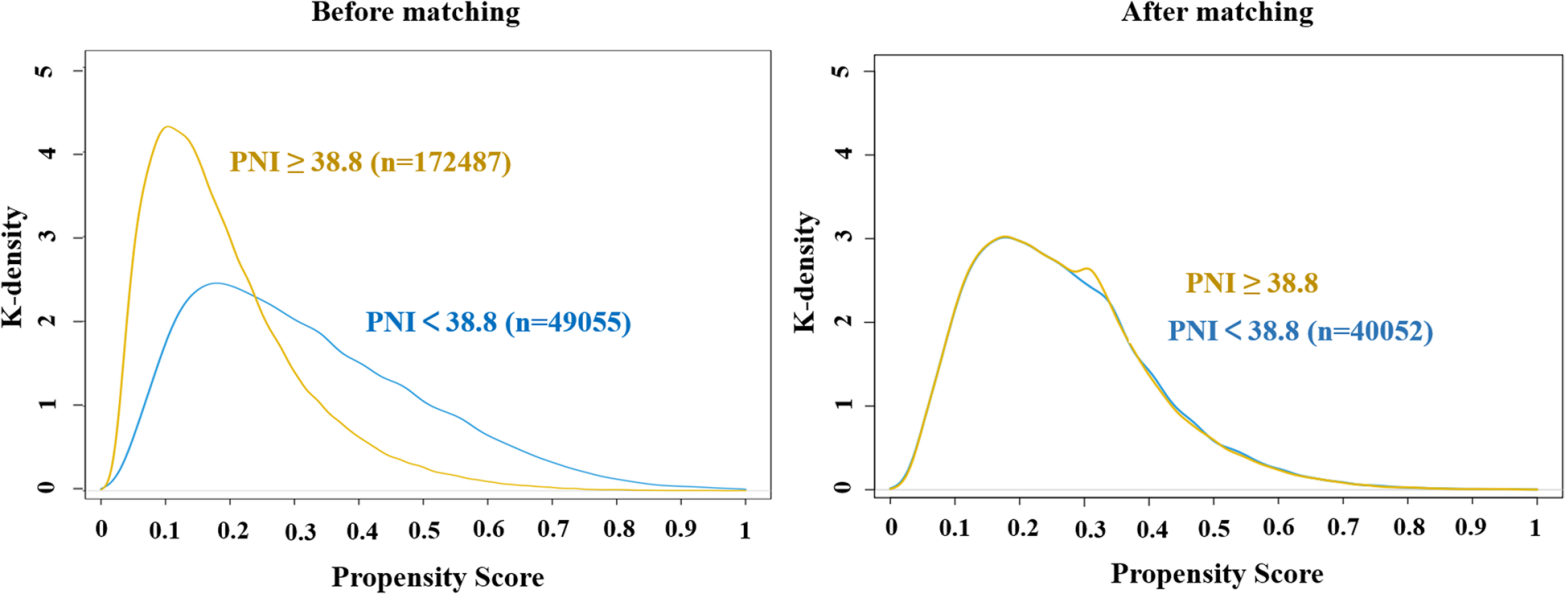
Distribution of propensity scores before and after matching

We performed further PSM analysis to omit unmatched variables. In the PSM cohort study, we matched 15 variables between PNI ≥ 38.8 and PNI < 38.8 groups which are shown in Supplementary Table 2. A total of 80,106 patients in the PNI ≥ 38.8 group were matched at a ratio of 2:1 with 40,052 patients in the PNI < 38.8 group. The distribution of propensity scores of patients before and after PSM is shown in Fig. 2. After matching, the baseline characteristics of the two groups were generally well balanced, with SMDs less than 0.10 for most of the covariates. In the univariate logistic regression analysis after PSM, the OR of the PNI < 38.8 group was 1.250 (95% CI: 1.000–1.556, *P* = 0.050). Following multivariate logistic regression adjustment after PSM, the OR significantly increased to 1.357 (95% CI: 1.077–1.704, *P* = 0.009), while the association between PNI and perioperative ischemic stroke remained robust (Supplementary Table 2).

### Subgroup analyses

**Fig. 3:**
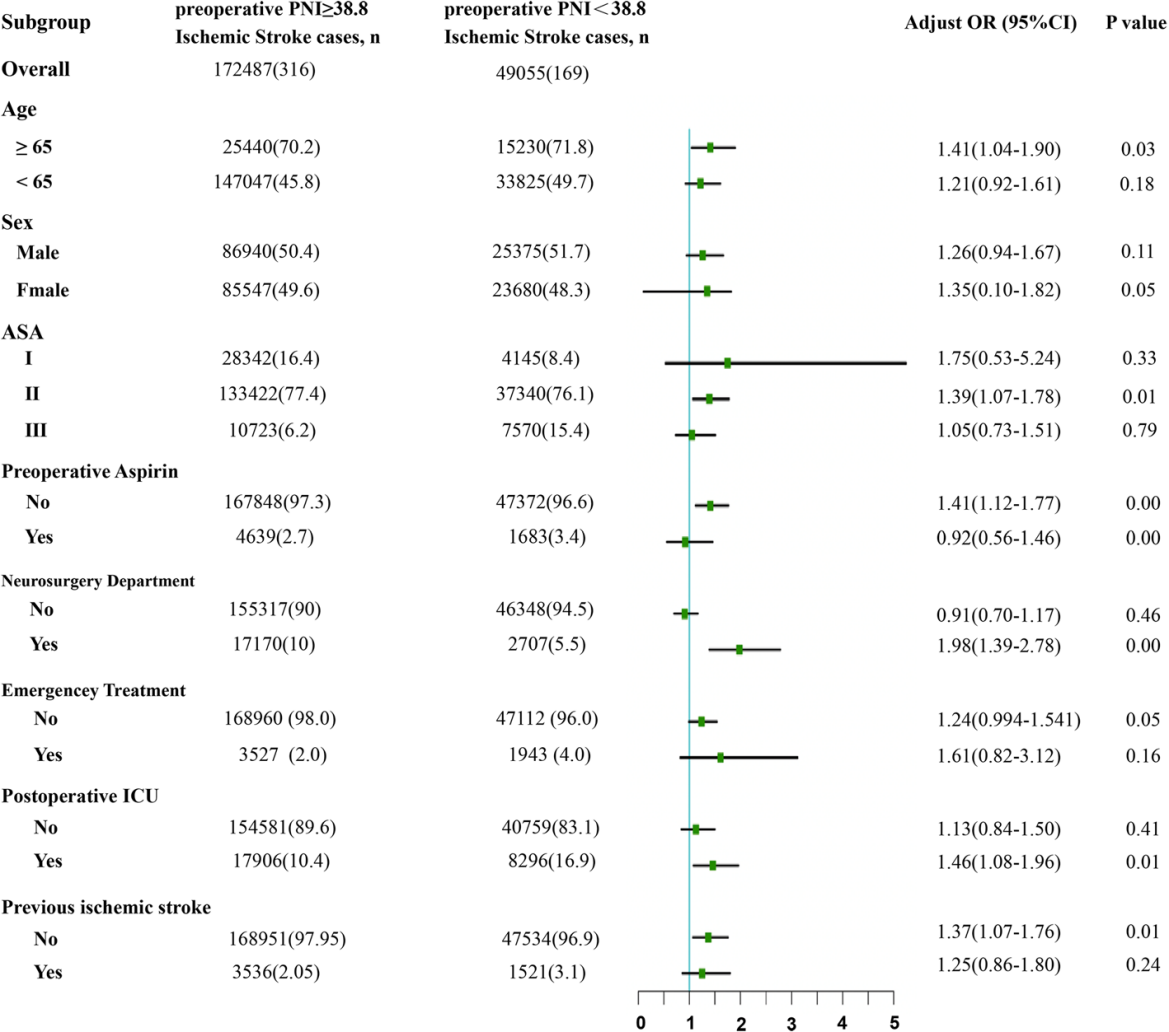
Subgroup analyses of the association between PNI and perioperative ischemic stroke. PNI, prognostic nutritional index; OR, odds ratio; CI, Confidence interval; ASA, American Society of Anesthesiologists classification

In our subgroup analyses, the reduced PNI was significant associated to an increased risk of perioperative ischemic stroke in patients for age ≥ 65 years old (OR = 1.41, 95% CI: 1.04–1.90, *P* = 0.03), female (OR = 1.35, 95% CI: 0.10–1.82, *P* = 0.05), and ASA II (OR = 1.39, 95% CI: 1.07–1.78, *P* = 0.01). In addition, the reduced PNI was significantly associated with perioperative ischemic stroke in patients who were not taking aspirin before surgery (OR = 1.41, 95% CI: 1.12–1.77, *P* = 0.00), didn’t have a history of stroke (OR = 1.24, 95% CI: 0.86–1.79, *P* = 0.01), and had neurosurgery (OR = 1.98, 95% CI: 1.39–2.78, *P* = 0.00) and non-emergency surgery (OR = 1.24, 95% CI: 0.994–1.541, *P* = 0.05). This increased risk was also significant in patients admitted to the ICU after surgery (OR = 1.46, 95% CI: 1.08–1.96, *P* = 0.01) (Fig. 3).

## Discussion

In this study the PNI cut-off value is determined to be 38.8 for predicting perioperative ischemic stroke risk in patients after non-cardiac surgery. The cut-off value for LLR, which had a different significance level between the PNI ≥ 38.8 group and the PNI < 38.8 group, was found to be 3.28. Our analysis suggests that reduced preoperative PNI is an important nutritional risk factor for perioperative ischemic stroke.

We first used univariate logistic analysis to investigate the association between perioperative ischemic stroke and PNI as categorical variable with a cut-off value of 38.8, and PNI < 38.8 was a significant predictor for perioperative ischemic stroke (OR = 1.884, 95% CI: 1.559—2.267, *P* < 0.001). We then performed a multivariate logistic regression analysis with a model including preoperative, intraoperative, and other variables, to further explore the association between perioperative ischemic stroke and PNI. In a similar way, the results revealed PNI < 38.8 to be an independent nutritional risk factor for ischemic stroke in patients after surgery (OR = 1.306, 95% CI: 1.061—1.602, *P* = 0.011). Though the studies reported that the risk for perioperative stroke depends on type of surgery and previous medical history [17, 20], we did not exclude patients with previous/recent cerebrovascular events and select type of surgery, the reason is that we want to prove that preoperative PNI is an independent risk factor useful to predict perioperative ischemic stroke risk in the whole population. To eliminate the potential residual confounding variables, we subsequently performed PSM analysis to further identify the relationship between perioperative ischemic stroke and PNI using univariate and multivariate logistic regression analyses. After matching, the results revealed that reduced PNI was significantly associated with an increased risk of perioperative ischemic stroke. In our subgroup analysis we found that PNI < 38.8 is a powerful nutritional predictor of perioperative ischemic stroke in patients of age ≥ 65 years old, ASA II, not taking aspirin before surgery, without a history of stroke, who had neurosurgery, non-emergency surgery, and were admitted to ICU after surgery.

Chronic inflammation plays a vital role in the pathology and development of ischemic stroke. PNI, an index calculated using the serum albumin and lymphocyte count, is a biological marker that indicates a patient’s chronic inflammatory and nutritional status [6]. Serum albumin, a unique multifunctional protein, is reported to exert significantly neuroprotective effects against ischemic stroke via reducing the hematocrit level and inhibiting oxidizing agents [21, 22]. An important clinical trial published in *Stroke* found that relatively low serum albumin level is associated with poor outcome in patients with ischemic stroke [23]. Lymphocytes are also known to play a pivotal role in ischemic stroke [24], and one study showed that lower lymphocyte counts correlate with poor functional outcomes after acute ischemic stroke [25]. LLR is associated with inflammatory processes and believed to be involved in the pathogenesis of ischemic stroke [15, 26]. Taking into account that LLR ≥ 3.28 is significantly associated with the increase of perioperative ischemic stroke in univariate analysis and LLR is considered to be involved in the development of ischemic stroke, we included LLR in multivariate analysis in the study, and further identified that the decreased PNI is an independent risk factor to predict perioperative ischemic stroke. Therefore, a decreased PNI value indicates that patients are in a malnourished and inflammatory status, which has the potential to become a risk predictor for perioperative ischemic stroke.

To our knowledge this is the first study to evaluate the association between the preoperative PNI and ischemic stroke in patients with non-cardiac surgery. Screening the nutritional status plays a vital role in patients with perioperative ischemic stroke after non-cardiac surgery. However common malnutrition screening tools, such as the malnutrition universal screening tool for malnutrition, may be unsuitable for use in clinical practice due to complex measurement procedures and the need for professional assistance [27]. PNI could be used as a screening tool for nutritional status in clinical settings, as it can be easily calculated from parameters that are routinely measured in the case record: serum albumin concentration and lymphocytes count. We believe, given our incorporation in the study of data from a large-scale database, that the experimental results are relatively accurate. In our study we observed that decreased PNI was an independent predictor of perioperative stroke. These results suggest that preoperative PNI may be a biological marker to predict perioperative ischemic stroke risk.

Previous studies have reported that poor nutrition increases the risk of ischemic stroke [28–30], and a recent randomized clinical trial of medically hospitalized patients at nutritional risk found that the use of individualized nutritional support during hospitalization improved important clinical outcomes, including survival, compared to standard hospital food [31]. Since preoperative PNI is a biological marker that reflects a patient’s nutritional status, we could intervene and provide treatments —treating the preoperative underlying diseases (hypertension, diabetes, atrial tremor, etc.), enhancing care, individualizing nutritional support for patients before surgery to prevent perioperative ischemic stroke— to improve the preoperative PNI. Preoperative PNI could be used as an intervenable preoperative clinical biochemical index to reduce the incidence of perioperative ischemic stroke. That is, PNI < 38.8 was significantly associated with an increased incidence of perioperative ischemic stroke in patients undergoing non-cardiac surgery. Monitoring the PNI indicators is helpful to predict the occurrence of malnutrition before operation, so as to provide guidance for the prevention of malnutrition and reduce the incidence of perioperative ischemic stroke in patients. Addressing poor nutrition requires collaborative efforts across various sectors, including healthcare providers, nursing, and availability of nutritious food. However further studies including prospective studies are required to help verify the preoperative PNI is an intervenable clinical indicator. Future research is crucial to advance the understanding and improvement of the preoperative PNI.

We identified some limitations in our study: firstly, our study was conducted on a retrospective cohort in a single institute, which may have generated biases for the differences between groups and variability in diagnostic testing. Secondly, an evaluation was made between the preoperative laboratory parameters and the incidence of perioperative ischemic stroke, but there are other factors such as frailness and sarcopenia which have not been considered in this study, and could be taken into account to evaluate the nutritional status. Lastly, we were not able to determine the relationship between the specified parameters and the nutritional therapy given to the patients. Thus we recommend further studies to assess these issues.

Our study shows that low preoperative PNI is significantly associated with a higher incidence of ischemic stroke in patients undergoing non-cardiac surgery. Preoperative PNI, a preoperative nutritional status evaluation index, is an independent risk factor to assess perioperative ischemic stroke risk, and could be used as an intervenable preoperative clinical biochemical index to reduce the incidence of perioperative ischemic stroke.

## Conclusion

The preoperative PNI is an independent factor to predict perioperative ischemic stroke risk. PNI may be a biological marker to assess a patient’s chronic inflammatory and nutritional status, and it could be a useful predictive information and intervention target on perioperative ischemic stroke in non-cardiac surgery patients.

### Supplementary Information

**Additional file 1:** **Fig. S1.** ROC curve of PNI for perioperative ischemic stroke. ROC, receiver operating characteristics curve; PNI, prognostic nutritional index. **Fig. S2.** ROC curve of LLR for perioperative ischemic stroke. ROC, receiver operating characteristics curve; LLR, leucocyte-to-lymphocyte ratio. **Table 1.** Univariate and multivariate logistic regression analyses for perioperative ischemic stroke in the Model 1 and Model 2. **Table 2.** Univariate and multivariate logistic regression analyses for perioperative ischemic stroke in the PS matching.

## Data Availability

The data supporting the findings of this study are available on request from the corresponding author.

## Abbreviations

OR: Odds ratio
CI: Confidence interval
PNI: Prognostic nutritional index
SMD: Standardized mean difference
PSM: Propensity Score Matching
BMI: Body mass index
COPD: Chronic obstructive pulmonary disease
ASA: American Society of Anaesthesiologists classification system
ACEI: Angiotensin-converting enzyme inhibitors
ARB: Angiotensin receptor blockers
LLR: Leucocyte-to-lymphocyte ratio
E.N.T: Otolaryngology head, and neck surgery
MAP: Mean arterial pressure
NSAIDs: Non-steroidal anti-inflammatory drugs
