# Supplementary material for: Prognostic Nutritional Index (PNI) as a potential predictor and intervention target for perioperative ischemic stroke: a retrospective cohort study
Source: BMC Anesthesiol. 2023 Aug 10;23:268. doi: 10.1186/s12871-023-02216-8 (PMC10413636; doi:10.1186/s12871-023-02216-8)
Supplement: Supplementary file 1 — Additional file 1: Fig. S1. ROC curve of PNI for perioperative ischemic stroke. ROC, receiver operating characteristics curve; PNI, prognostic nutritional index. Fig. S2. ROC curve of LLR for perioperative ischemic stroke. ROC, receiver operating characteristics curve; LLR, leucocyte-to-lymphocyte ratio. Table 1. Univariate and multivariate logistic regression analyses for perioperative ischemic stroke in the Model 1 and Model 2. Table 2. Univariate and multivariate logistic regression analyses for perioperative ischemic stroke in the PS matching. [file 12871_2023_2216_MOESM1_ESM.doc]

Supplemental Materials

**Supplementary Figure 1**. ROC curve of PNI for perioperative ischemic stroke. ROC, receiver operating characteristics curve; PNI, prognostic nutritional index.

**
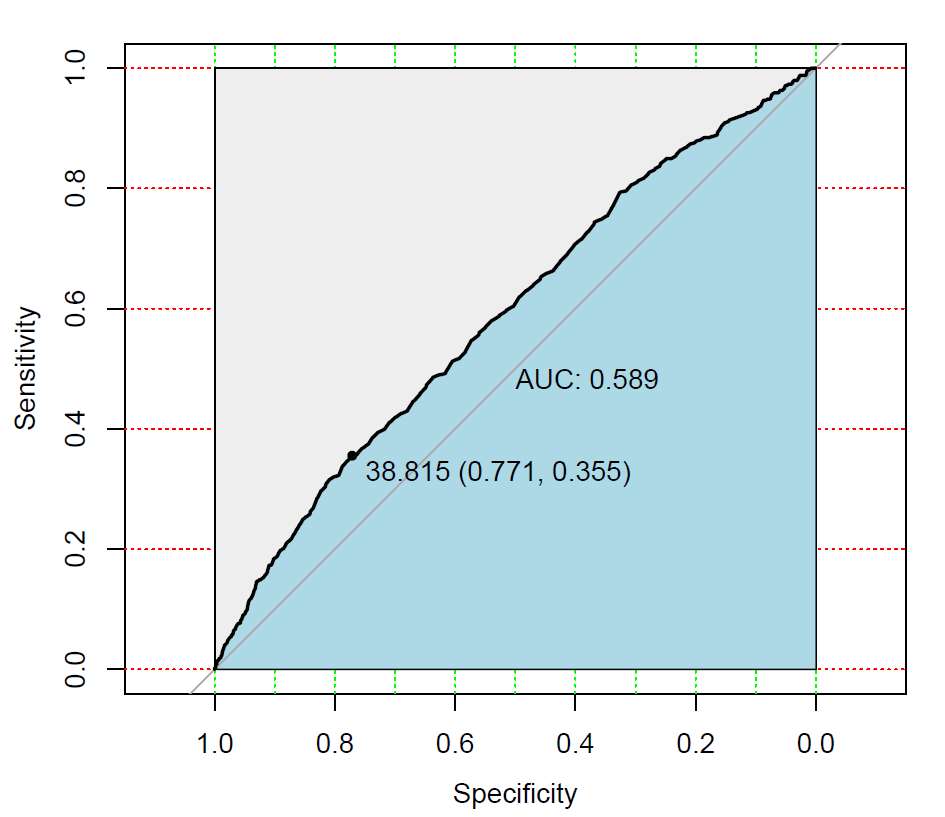
**

**Supplementary Figure 2**. ROC curve of LLR for perioperative ischemic stroke. ROC, receiver operating characteristics curve; LLR, leucocyte-to-lymphocyte ratio.

**
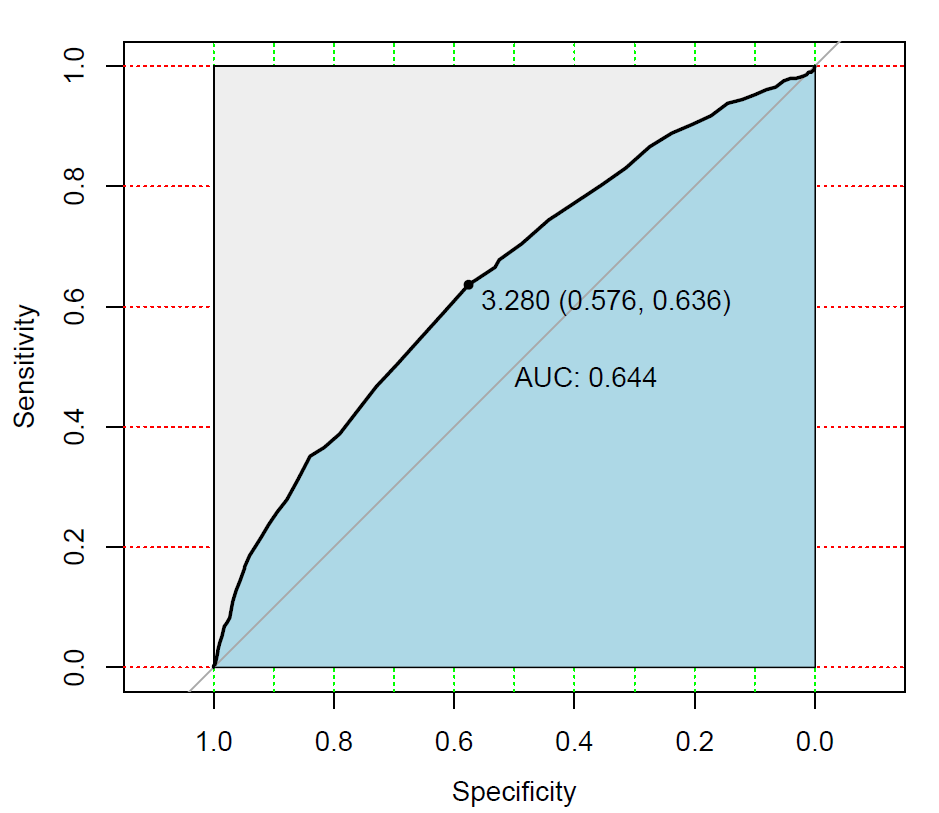
**

**Supplementary Table 1** Univariate and multivariate logistic regression analyses for perioperative ischemic stroke in the Model 1 and Model 2.

| Variables | Univariate analysis | | Multivariate analysis | |
| --- | --- | --- | --- | --- |
|  | OR (95% CI) | *P* value | OR (95% CI) | *P* value |
| PNI (< 38.8 vs ≥38.8) | 1.884 (1.559-2.267) | < 0.001 | 1.306 (1.061–1.602) | 0.011 |
| Age | 1.06(1.060-1.100) | < 0.001 | 1.044 (1.035–1.053) | < 0.001 |
| Sex (female vs male) | 0.940(0.790-1.000) | 0.520 | 1.216 (1.001–1.474) | 0.048 |
| Hypertension (Yes vs No) | 4.280(3.580-5.000) | < 0.001 | 1.276 (1.033–1.576) | 0.024 |
| Diabetes mellitus (Yes vs No) | 3.040(2.500-4.000) | < 0.001 | 1.606 (1.301–1.972) | < 0.001 |
| Prior ischemic stroke (Yes vs No) | 20.310(16.740-25.000) | < 0.001 | 7.308 (5.879–9.068) | < 0.001 |
| β-blockers medication (Yes vs No) | 3.640(2.770-5.000) | < 0.001 | 1.411 (1.041–1.880) | 0.022 |
| Aspirin medication (Yes vs No) | 9.430(7.580-12.000) | < 0.001 | 2.659 (2.072–3.388) | < 0.001 |
| Preoperative glucocorticoid (Yes vs No) | 1.610(1.210-2.000) | < 0.001 | 1.498 (1.092–2.016) | 0.010 |
| Preoperative MAP | 1.050(1.000-1.050) | < 0.001 | 1.022 (1.014–1.030) | < 0.001 |
| Preoperative LLR | 1.050(1.000-1.040) | < 0.001 | 1.007 (0.985–1.024) | 0.504 |
| Preoperative LLR (≥ 3.28 vs ＜3.28) | 2.390(1.980-3.000) | < 0.001 | 1.475 (1.200–1.816) | < 0.001 |
| Facility (selective vs emergency) | 4.680(3.490-6.000) | < 0.001 | 0.588 (0.412–0.853) | 0.004 |
| Surgical procedures |  |  |  |  |
| Trauma surgery | 0.530(0.230-1.000) | 0.140 | 0.195 (0.074–0.428) | < 0.001 |
| Gynaecology | 0.450(0.240-1.000) | 0.010 | 0.474 (0.237–0.874) | 0.024 |
| Intra-abdominal surgery | 0.630(0.450-1.000) | 0.010 | 0.266 (0.184–0.388) | < 0.001 |
| Joint arthroplasty | 1.640(1.110-2.000) | 0.010 | 0.666 (0.434–1.018) | 0.061 |
| Spine | 1.820(1.250-3.000) | < 0.001 | 0.913 (0.613–1.360) | 0.652 |
| Urinary surgery | 0.750(0.460-1.000) | 0.240 | 0.415 (0.250–0.670) | < 0.001 |
| Neurosurgery | 0.490(0.180-1.000) | 0.170 | 1.063 (0.724–1.578) | 0.757 |
| Thoracic or vascular | 4.980(3.670-7.000) | < 0.001 | 0.455 (0.286–0.712) | 0.001 |
| Other (plastic surgery, etc.) | 1.070(0.690-2.000) | 0.770 | 0.465 (0.140–1.150) | 0.144 |
| Bleeding volume |  |  |  |  |
| (50,200] | 1.720(1.370-2.000) | < 0.001 | 1.305 (1.031–1.658) | 0.028 |
| ＞200 | 2.210(1.740-3.000) | < 0.001 | 1.735 (1.336–2.259) | < 0.001 |
| ICU admission after surgery (Yes vs No) | 6.720(5.620-8.000) | < 0.001 | 3.118 (2.387–4.069) | < 0.001 |
| Abbreviations: PNI, prognostic nutritional index; MAP, mean arterial pressure; LLR, leucocyte-to-lymphocyte ratio. | | | | |

**Supplementary Table 2** Univariate and multivariate logistic regression analyses for perioperative ischemic stroke in the PS matching.

| Variables | Univariate analysis | | Multivariate analysis | |
| --- | --- | --- | --- | --- |
|  | OR (95% CI) | *P* value | OR (95% CI) | *P* value |
| PNI (< 38.8 vs ≥38.8) | 1.250(1.000-1.556) | 0.050 | 1.357 (1.077-1.704) | 0.009 |
| Age | 1.060(1.000-1.050) | < 0.001 | 1.040 (1.029-1.051) | < 0.001 |
| Sex (female vs male) | 0.850(0.690-1.000) | 0.140 | 1.062 (0.837-1.344) | 0.620 |
| Hypertension (Yes vs No) | 3.490(2.820-4.000) | < 0.001 | 1.154 (0.893-1.489) | 0.273 |
| Diabetes mellitus (Yes vs No) | 2.540(2.010-3.000) | < 0.001 | 1.563 (1.207-2.008) | 0.001 |
| Prior ischemic stroke (Yes vs No) | 17.770(14.050-22.000) | < 0.001 | 7.463 (5.726-9.700) | < 0.001 |
| β-blockers medication (Yes vs No) | 3.790(2.770-5.000) | < 0.001 | 1.650 (1.155-2.309) | 0.005 |
| Aspirin medication (Yes vs No) | 8.290(6.390-11.000) | < 0.001 | 2.690 (1.995-3.591) | < 0.001 |
| Preoperative glucocorticoid (Yes vs No) | 1.480(1.050-2.000) | 0.030 | 1.494 (1.014-2.139) | 0.035 |
| Preoperative MAP | 1.050(1.000-1.040) | < 0.001 | 1.021 (1.011-1.030) | < 0.001 |
| Preoperative LLR | 1.050(1.000-1.040) | < 0.001 | 0.995 (0.960-1.025) | 0.747 |
| Preoperative LLR (≥ 3.28 vs ＜3.28) | 1.930(1.540-2.000) | < 0.001 | 1.381 (1.070-1.79) | 0.014 |
| Facility (selective vs emergency) | 0.220(0.000-0.160) | < 0.001 | 0.542 (0.350-0.857) | 0.007 |
| Surgical procedures |  |  |  |  |
| Trauma surgery | 0.380(0.150-1.000) | 0.040 | 0.276 (0.093-0.653) | 0.008 |
| Gynaecology | 0.280(0.120-1.000) | < 0.001 | 0.373 (0.149-0.811) | 0.021 |
| Intra-abdominal surgery | 0.340(0.220-1.000) | < 0.001 | 0.237 (0.151-0.376) | < 0.001 |
| Joint arthroplasty | 1.220(0.780-2.000) | 0.390 | 0.717 (0.442-1.168) | 0.179 |
| Spine | 1.290(0.820-2.000) | 0.280 | 0.756 (0.462-1.238) | 0.266 |
| Urinary surgery | 0.560(0.310-1.000) | 0.060 | 0.367 (0.193-0.666) | 0.001 |
| Neurosurgery | 0.280(0.070-1.000) | 0.080 | 0.945 (0.59-1.536) | 0.815 |
| Thoracic or vascular | 4.720(3.260-7.000) | < 0.001 | 0.509 (0.297-0.859) | 0.012 |
| Other (plastic surgery, etc.) | 0.980(0.590-2.000) | 0.930 | 0.329 (0.053-1.091) | 0.128 |
| Bleeding volume |  |  |  |  |
| (50,200] | 1.710(1.270-2.000) | < 0.001 | 1.450 (1.065-1.995) | 0.020 |
| ＞200 | 2.350(1.740-3.000) | < 0.001 | 2.249 (1.621-3.147) | < 0.001 |
| ICU admission after surgery (Yes vs No) | 6.540(5.270-8.000) | < 0.001 | 3.421 (2.462-4.729) | < 0.001 |
| Abbreviations: PNI, prognostic nutritional index; MAP, mean arterial pressure; LLR, leucocyte-to-lymphocyte ratio. | | | | |
